# Development and Delphi consensus validation of the Medication-Related Fall screening and scoring tool

**DOI:** 10.1007/s11096-024-01734-w

**Published:** 2024-05-16

**Authors:** Dima Saeed, Gillian Carter, Ruth Miller, Carmel Darcy, Karen Miller, Kevin Madden, Hilary McKee, Jayne Agnew, Paula Crawford, Carole Parsons

**Affiliations:** 1 School of Pharmacy, Queen’s University Belfast, Belfast, UK; 2 School of Pharmacy, Middle East University, Amman, Jordan; 3 School of Nursing and Midwifery, Queen’s University Belfast, Belfast, UK; 4 Western Health and Social Care Trust, Londonderry, UK; 5 South Eastern Health and Social Care Trust, Belfast, UK; 6 Northern Health and Social Care Trust, Antrim, UK; 7 Southern Health and Social Care Trust, Craigavon, UK; 8 Belfast Health and Social Care Trust, Belfast, UK

**Keywords:** Assessment tool, Fall-risk increasing drugs, Falls, Medication review, Older people, Polypharmacy

## Abstract

**Background:**

Falls are a significant public health problem and constitute a major cause of injuries and mortality. Risk factors for falls are multifactorial and include medication use.

**Aim:**

To develop and investigate the content validity of the Medication-Related fall (MRF) screening and scoring tool.

**Method:**

The MRF tool was developed from clinical practice guidelines addressing medication-related problems, and additional medications identified by specialist pharmacists across a region of the United Kingdom (Northern Ireland). Medication classes were categorised according to their ‘potential to cause falls’ as: high-risk (three points), moderate-risk (two points) or low-risk (one point). The overall medication-related falls risk for the patient was determined by summing the scores for all medications. The MRF was validated using Delphi consensus methodology, whereby three iterative rounds of surveys were conducted using SurveyMonkey^®^. Twenty-two experts from 10 countries determined their agreement with the falls risk associated with each medication on a 5-point Likert scale. Only medications with at least 75% of respondents agreeing or strongly agreeing were retained in the next round.

**Results:**

Consensus was reached for 19 medications/medication classes to be included in the final version of the MRF tool; ten were classified as high-risk, eight as moderate-risk and one as low-risk.

**Conclusion:**

The MRF tool is simple and has the potential to be integrated into medicines optimisation to reduce falls risk and negative fall-related outcomes. The score from the MRF tool can be used as a clinical parameter to assess the need for medication review and clinical interventions.

## Impact statetments

- The MRF tool is an informative tool that can be used in different healthcare settings to evaluate and optimise medications to reduce falls and fall-related negative outcomes.
- The MRF tool can help to standardise clinical practice and guidelines on medication-related falls and promote awareness and knowledge among healthcare professionals on this topic.
- The MRF score can be used as a clinical parameter to assess the need for medication review and clinical interventions.

## Introduction

Falls are a significant public health problem worldwide and constitute a major cause of injuries and injury-related admissions to hospital, emergency department visits and mortality as well as increased healthcare resource utilisation and cost [1–3]. Risk factors for falls are multifactorial and may include age, gender, or length of hospital stay [2]. It is well established that medication use is a risk factor for falls [4–6]. Fall-risk-increasing drugs (FRIDs) are widely prescribed for older people [6–10]. A strategy to avoid and reduce FRID use represents an essential component of a multifactorial falls risk management approach and should be implemented in routine practice in healthcare settings [6, 20, 21] to reduce the incidence of falls [6, 10–19, 22].

Tools used to address potentially inappropriate prescribing such as the Beers Criteria [23], the STOPP/START Criteria [24] and the Medication Appropriateness Index (MAI) [25], have been considered to be overly arduous for falls-risk screening and lack specificity. Although the STOPPFall (Screening Tool of Older Persons Prescriptions in Older Adults with High Fall Risk) tool was developed recently using the Delphi approach [26], it does not use a scoring system to facilitate integration of falls risk assessment into holistic medication review and use in clinical practice to optimise medications. In the context of limited healthcare resources and increasing demand, a rapid scoring tool is needed to facilitate identification of patients for medication review based on their risk of medication-related problems.

NHS Scotland published polypharmacy guidelines to address medication-related problems resulting from multimorbidity [27]. These list commonly prescribed medication classes categorised into high, moderate or low risk in terms of their ‘potential to cause falls’. This list has recently been used by pharmacists to assess the medication-related falls and to optimise medication prescribing for frail older patients in an acute care frailty ward in in a region of the United Kingdom (UK) [Northern Ireland (NI)][28]. However, this list has not been validated as a falls-risk screening instrument.

### Aim

This study aimed to develop and investigate the content validity of the Medication-Related fall (MRF) screening and scoring tool.

### Ethics approval

This study received ethical approval from the Faculty of Medicine, Health and Life Sciences Research Ethics Committee, Queen’s University Belfast on 10th September 2020 (MHLS 20_102).

## Method

**Fig. 1:**
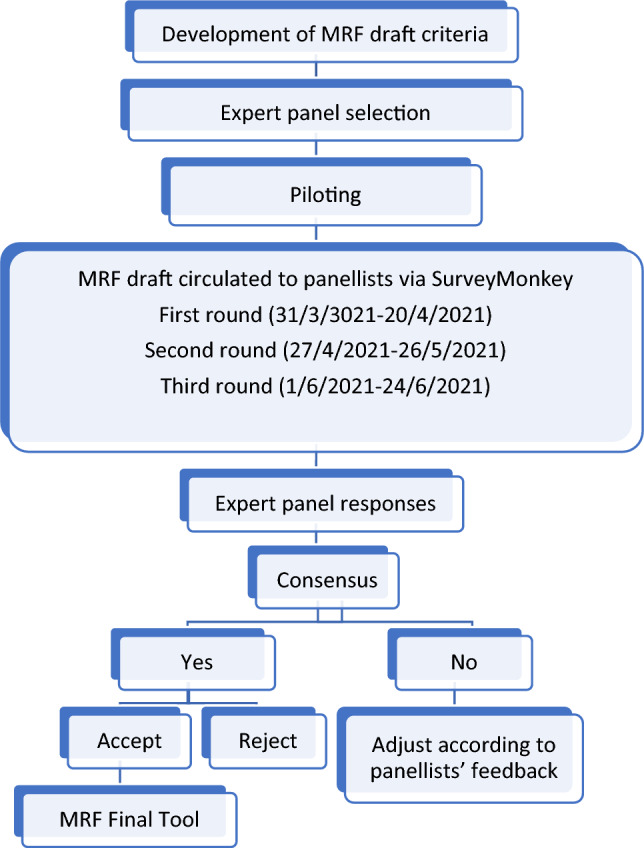
Description of the Delphi approach and study timelines

This study comprised four stages, as described in Fig. 1.

Content validation is the first step of rational instrument development [29]. The Delphi consensus validation approach [30] was used to develop and investigate the content validity of the MRF tool. This approach is considered to be particularly appropriate in determining content validity as the consensus panel participating in the study are representative of the group or the area of knowledge [31]. It offers anonymity and confidentiality, which prevents domination by leading individuals and pressure from the group [32]. Anonymity may also encourage openness to express honest opinions, and multiple iterative rounds allow participants to re-evaluate their ideas leading to confirmation and increased content validity [32, 33]. This method has been used to develop and determine the content validity of a variety of widely accepted prescribing assessment tools such as the STOPP/START criteria Versions 1, 2 and 3, the FORTA (Fit FOR The Aged) tool, and STOPPFrail Versions 1 and 2 [24, 34–38].

### Development of MRF draft criteria

The initial draft of the MRF tool included the medicines listed as causing falls in the NHS Scotland polypharmacy guidelines [27]. The tool also included medications associated with increased risk of falls identified by medicines optimisation for older people (MOOP) pharmacists (with recognised expertise in geriatric medicine and pharmacotherapy in older people) as having a falls risk evident from their mechanism of action or side effects or from sources such as the British Geriatrics Society Falls Guidance [22] and Beers Criteria [39] and educational sessions on polypharmacy and falls. Medications added by the MOOP team were assigned potential falls risk categories based on assessments by five pharmacists throughout all five Health and Social Care Trusts (main administrative health areas) in NI. The questionnaire for the first Delphi round was divided into four sections. Section A collected demographic data (e.g. highest level of education and clinical area of expertise). Sections B, C, and D presented high-risk, moderate-risk and low-risk medications, respectively, as determined from the NHS Scotland polypharmacy guidelines [27] or by the specialist and consultant pharmacists. In each section, each criterion was presented in the form of a statement, i.e. a medication/medication class followed by details regarding the mechanism by which each medication/medication class may increase falls risk. Questionnaires for the second and third rounds did not collect demographic data, and were therefore divided into three sections presenting high-risk, moderate-risk and low-risk medications.

High-risk medication classes were defined as those medications that *may commonly cause or contribute to falling risk* on their own or in combination, and were selected based on evidence from observational studies and clinical experience suggesting these medications as “high-risk” for causing falls. Moderate-risk medication classes were defined as those which *may cause falls* especially in combination. Studies and systematic reviews have reported conflicting results regarding the association between the use of moderate-risk medications and risk of falling. However, falls-related side-effects are possible, and using these medications in combination may increase risk of falling. Low-risk medication classes were defined as those that *possibly cause falls*, particularly in combination. Published evidence of a direct link between these medications and an increase in falls risk is lacking. The definitions of high, moderate and low risk were stated in each round of the survey.

To provide a ranking and scoring system for medication-related falls risk, high-risk medications were assigned a score of three points, moderate-risk medications a score of two and low-risk medications a score of one. When a drug fell into more than one medication class, the highest score was assigned. The overall medication-related falls risk was determined by summing these scores.

### Recruitment of the expert panel

In March 2021, 72 experts were invited via email to participate. Experts were defined as individuals with recognised expertise in geriatric medicine and pharmacotherapy in older people, such as geriatricians, clinical pharmacologists, clinical pharmacists, old age psychiatrists, general practitioners, researchers, and university academics. Explicit selection criteria were: (i) senior-level experience in pharmacotherapy i.e. five or more years; (ii) published work in international peer-reviewed journals within the previous five years; and (iii) professor/senior lecturer/consultant status in the relevant discipline. Twenty-two experts across 10 countries agreed to participate (Table 1).

**Table 1:** Characteristics of Delphi panel members

Description	No. of experts (% of total)
**Country**	
United Kingdom	7 (31.8)
Canada	5 (22.7)
USA	2 (9.1)
Ireland	2 (9.1)
Australia	1 (4.5)
Belgium	1 (4.5)
Sweden	1 (4.5)
Malaysia	1 (4.5)
Turkey	1 (4.5)
France	1 (4.5)
**Current employment**	
Academic/researcher	10 (45.5)
Clinical academic doctor	5 (22.7)
Doctor	4 (18.2)
Pharmacist	2 (9.1)
Clinical academic pharmacist	1 (4.5)
**Length of experience in current employment**	
1–5 years	2 (9.1)
6–10 years	4 (18.2)
11–15 years	2 (9.1)
More than 15 years	14 (63.6)
**Current place of employment**	
GP surgery/healthcare centre	1 (4.5)
Hospital	8 (36.4)
Primary care, intermediate care, healthcare interface	2 (9.1)
College/University	8 (36.4)
Geriatric day hospital (as a clinician), and research institute	1 (4.5)
Joint clinical appointment	1 (4.5)
Retired	1 (4.5)
**Highest level of education**	
PhD	11 (50.0)
MSc	1 (4.5)
MD	4 (18.2)
Doctor of pharmacy	2 (9.1)
Graduate degree/university degree/graduate school and professional degree	4 (18.2)

### Piloting

The initial draft was piloted with three experts, who met the inclusion criteria for the main panellists, to ensure that the MRF tool and the Delphi consensus approach were clearly understood by panel members, and to determine the time required to complete the survey and any procedural problems associated with its completion.

### Survey distribution

The MRF tool was distributed to the expert panel, with three Delphi validation rounds conducted [30]. Further rounds were not conducted as it was considered that this could result in increased attrition and reduced response rates [40–42] and most studies typically use two or three rounds [43, 44].

Each round of the survey was sent to panellists for online completion using SurveyMonkey^®^ software. Panellists rated their agreement with the assigned falls-risk using a 5-point Likert scale, (Strongly agree = 5, Agree = 4, Neutral = 3, Disagree = 2, Strongly disagree = 1) [45]. Percentages for level of agreement, median Likert scale response and interquartile range (IQR) were determined for each statement. A median value of 4 or 5 with the 25th percentile (P_25_) of ≥ 4 was required for inclusion in the tool, i.e. only statements with at least 75% of respondents agreeing or strongly agreeing were included. Proposed criteria with a median value of 4 or 5 and a P_25_ of < 4 were modified as per panellists’ suggestions and included in the next Delphi round; those with a median value of ≤ 3 were excluded. Free-text spaces were available for suggestions or comments (including suggesting medications/medication classes not included in the MRF tool) as appropriate.

Following the first validation round, any proposed criteria that did not meet retention requirements were removed. The second and third rounds of the survey were created based on the panel comments and suggestions of the previous round. A summary of the group results and individual results after each round was sent to each panel member. Retained criteria that were modified, together with any new potential medications identified from the previous round, were included in the subsequent survey round. As in the first validation round, criteria that did not meet the retention requirements were excluded.

### Data analysis and management

All returned surveys were downloaded from Survey Monkey to a password-protected laptop computer and converted to a customised Microsoft Excel spreadsheet, which was exported to IBM SPSS^®^ Statistics Version 26 (IBM Corp., New York, USA) for analysis. The qualitative data (responses to the open-ended question) from each round were sorted by the research team to eliminate overlap or repetition and translated into a structured survey item in the following round. Any suggested medications were also added to the next round of the survey.

After the third round, only statements with a median value of 4 or 5 and the P_25_ of ≥ 4 were accepted for inclusion in the tool.

## Results

**Fig. 2:**
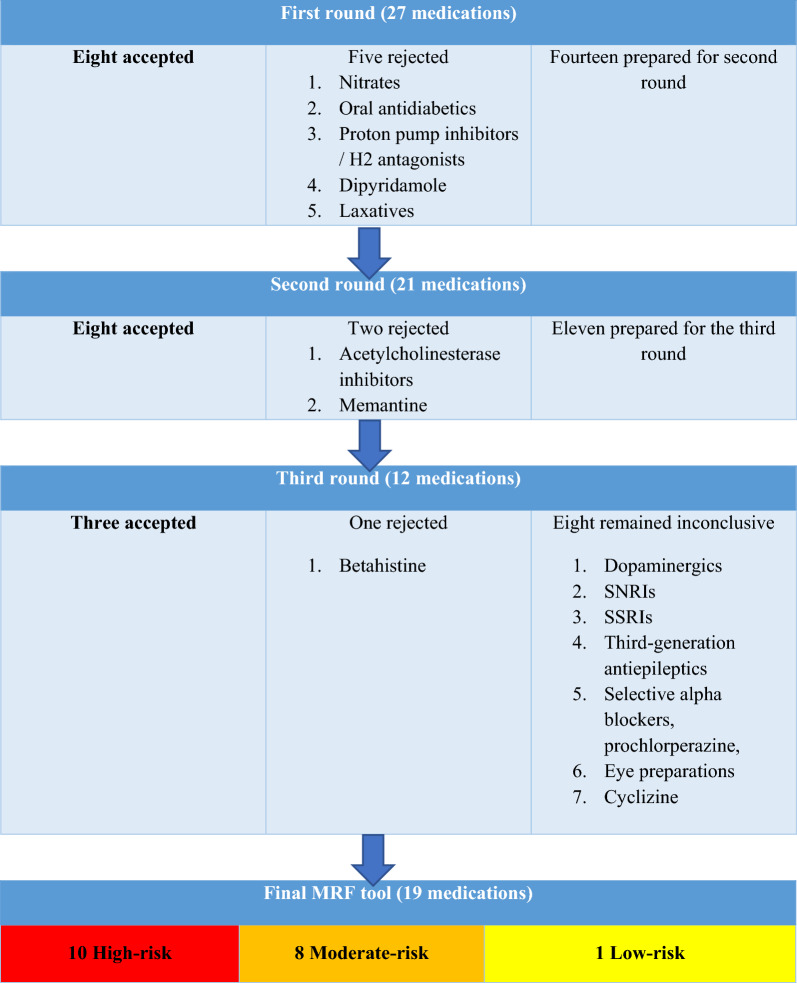
Summary of the results of the three iterative rounds of the survey

All 22 panellists completed all three rounds of the Delphi survey. Figure 2 details the numbers of medications accepted and rejected in each round.

After each round, the retained medications were modified to change assigned falls risk or split into different classes based on the panellists’ comments. After three iterative rounds, 19 medications/medication classes were included in the final version (Table 2).

**Table 2:**
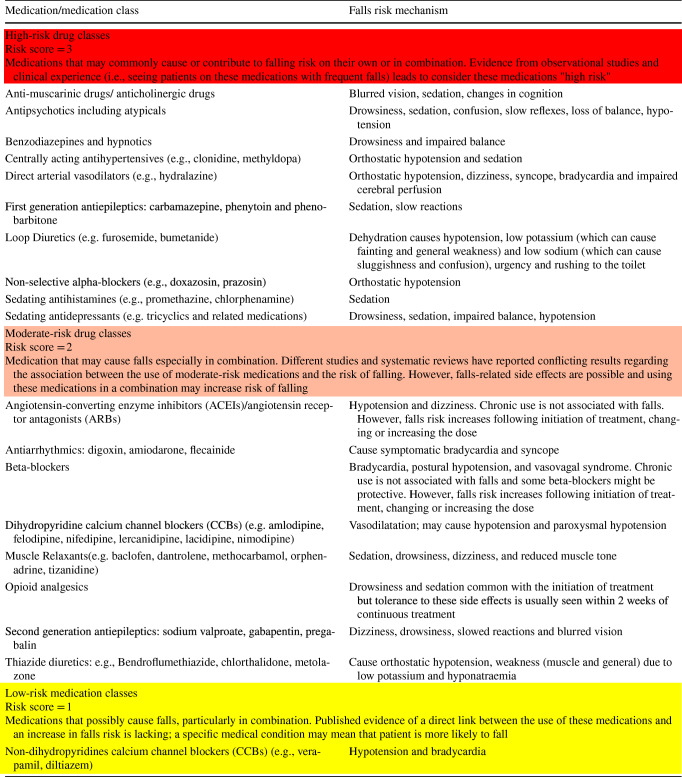
The MRF tool: final version

Consensus was not reached regarding serotonin and norepinephrine reuptake inhibitors (SNRIs), selective alpha-blockers, prochlorperazine, and cyclizine; these were therefore not included in the final version of the MRF tool. Although approximately three-quarters (77%) of the panel considered dopaminergic anti–Parkinson’s disease medications and selective serotonin reuptake inhibitors (SSRIs) as moderate-risk and eye preparations as low risk, these were not included in the tool because P_25_ was less than 4 (P_25_ = 3.8).

## Discussion

### Statement of key findings

The Medication-Related Fall (MRF) screening and scoring tool is an explicit list of 19 medications/medication classes that was developed and validated using Delphi consensus methodology.

### Evidence-driven tool

The high-risk category in the MRF tool includes medications/medication classes that act on the central nervous system and can cause sedation and drowsiness such as antidepressants, benzodiazepines, hypnotics, antipsychotics and anticholinergics. The association of these medications with falls has been consistently reported in previous studies including systematic reviews and meta-analyses [11–17, 23, 46–60].

Among the identified high-risk medications in the MRF tool are the medications that cause orthostatic hypotension i.e., centrally acting antihypertensives, direct arterial vasodilators and non-selective alpha-blockers. A systematic review and meta-analysis reported a significant and positive association between orthostatic hypotension and falls [61].

Regarding moderate-risk medications, many studies and systematic reviews have reported conflicting results concerning the association between the use of these medications and the risk of falling [15, 62]. This may indicate that the risk of falling associated with the use of these medications is patient-specific and may depend on the dose used, frequency, route of administration and the total drug regimen. This inconsistent association reported in the literature supports the classification of these medications into moderate-risk medications. The expert panel reached consensus in considering antihypertensive drugs (beta-blockers, angiotensin-converting enzyme inhibitors (ACEIs)/angiotensin receptors antagonists (ARBs), and dihydropyridine calcium channel blockers (CCBs)) as moderate-risk medication classes as they considered the risk of falls to be patient-specific (only for people who experience orthostatic hypotension), as well as dose and time-dependent. Chronic use was not deemed to be associated with the increased risk of falling. This is consistent with the findings of previous studies that the association between antihypertensive medications and risk of falling depends on the duration of use, an effect on cerebral perfusion, or a dose–effect [63, 64].

Loop diuretics were considered to be high-risk medications and thiazides to be moderate-risk medications in the final MRF tool. This is supported by a previous systematic review which reported that loop diuretics were the only pharmacological subgroup of diuretics that were significantly associated with the increased risk of falling [62].

In the MRF tool, first-generation anti-epileptics were classified as high-risk medications and second-generation anti-epileptics as moderate-risk medications; this may be explained by the more favourable adverse drug event (ADE) profile of second-generation anti-epileptics compared with first-generation agents [65]. Consensus was not reached regarding third-generation anti-epileptics; experts highlighted the lack of sufficient data to judge the association between these medications and risk of falling. A previous literature review also highlighted the limited data on side-effects of third-generation antiepileptics [66]. Furthermore, non-selective alpha-blockers were considered to be high-risk medications because they cause orthostatic hypotension [67]. However, consensus was not reached by the panel regarding selective alpha-blockers (e.g., tamsulosin). A previous single-blind randomised multicentre study reported that the incidence of dizziness and hypotension due to the administration of tamsulosin were significantly lower than that caused by terazosin, a non-selective alpha 1-adrenoceptor antagonist [68].

Consensus to accept or reject dopaminergic medications was not reached. This reflects the literature which reports that the association between dopaminergic medications and falls is difficult to quantify. In particular, falls are a key feature of advanced Parkinson’s disease [69].

### Comparison with available tools

Compared to STOPPFall [26], the MRF tool includes information about the mechanism associated with the increased risk of falling that could improve healthcare professionals’ knowledge of the risk of falls associated with therapeutic classes and individual medications as recommended by the European Geriatric Medicine Society (EUGMS) [70]. Furthermore, the MRF tool provides a ranking and scoring system to assess total falls risk associated with overall prescribing. The presence of a scoring system facilitates its integration into holistic medication reviews and its use in clinical practice to identify and prioritise patients most in need of medication review. The score can be used as a clinical parameter to assess prescribing decisions, similar to the MAI [25] and anticholinergic cognitive burden (ACB) scale [54], and its use by clinical pharmacists may enhance their integration into patient care in assessing and reducing falls risk. However, further studies are needed on the interpretation, predictive validity and threshold of risk of the overall MRF score.

The ‘ranking’ of medication classes with high- and moderate-falls-risk in the MRF tool is in line with a previous tool, the Medication Fall Risk Score (MFRS) system [71]. However, this system contains only nine medication classes and has not been widely adopted into practice or validated as a standalone screening instrument [72]

### Strengths and weaknesses

The MRF tool developed in this study is an evidence- and consensus-driven tool; the final medication list included is supported by published literature, including systematic reviews and meta-analyses. Many strategies were implemented to enhance its content validity. First, expert panel selection was based on explicit criteria to ensure the acquisition of experience, special skills, and in-depth knowledge. Second, a wide range of experts with academic and practical experience from geographically diverse locations was recruited into this study. Third, a systematic approach, a literature review, and the opinions and judgement of the experts were followed for drafting the first round. Fourth, conducting iterative rounds of the survey and controlled responses from subsequent rounds helped participants to evaluate, change, and/or develop their views. Fifth, anonymity was maintained through the iterative rounds enabling individual panel members to express their honest opinions confidentially without any social pressure. Finally, conducting the study online using data protection-compliant software facilitated participants’ responses and the dissemination of information from previous rounds.

Several limitations to this study should be acknowledged. Firstly, universally agreed definitions of consensus are lacking. However, ranges of between 51 and 75% have been suggested to demonstrate consensus [36, 37, 73]. In the present study, only statements to which at least 75% of the participants agreed or strongly agreed were included. Secondly, this study included three rounds, and consensus was not reached to include or exclude all medication classes. However, conducting further rounds was considered to put the process at risk of attrition of participants, and to constitute a competing threat to validity. Furthermore, agreement levels were high after the third round, and the comments provided by the experts indicated that there were few opportunities to reach consensus on the remaining eight medication classes.

### Interpretation and further research

The MRF tool has potential for integration into multifactorial risk assessments to identify patients at increased risk of falling, with the MRF total score used as a falls risk indicator informing prescribing decisions and medicines optimisation interventions. It can be used on hospital admission, when medications are changed and periodically as part of routine review. If patients are taking high- or moderate-risk medications, a referral for a medication review and fall prevention strategies should be prompted. The potential risk/benefit ratio of these medications should be assessed to decide whether to continue with these medications, to reduce the dose or to use an alternative medication or non-medication option. The MRF tool is currently integrated into the Pharmacist Falls Prevention service in NI to identify and prioritise those most at risk from medicines-related falls based on their MRF score and provide them with a medicines optimisation to reduce this risk. This has substantially reduced the waiting list for the highest-risk patients.

As content validity represents only one aspect of validity, future studies are needed to demonstrate predictive validity. Investigation of the accuracy, reliability, feasibility, and acceptability of the MRF tool must be conducted to further validate this tool.

## Conclusion

The MRF tool was developed using a consensus Delphi approach to evaluate and optimise medications to reduce falls risk and negative fall-related outcomes. It can help to standardise clinical practice and guidelines on medication-related falls and promote greater awareness and knowledge among healthcare professionals on this topic. The MRF tool score can be used as a clinical parameter to evaluate the appropriateness of prescribing and the need for medication review. Future investigation into the predictive validity and and reliability of the MRF tool must be conducted to further validate this tool.
